# Real-Time qPCR as a Method for Detection of Antibody-Neutralized Phage Particles

**DOI:** 10.3389/fmicb.2017.02170

**Published:** 2017-11-06

**Authors:** Anna Kłopot, Adriana Zakrzewska, Dorota Lecion, Joanna M. Majewska, Marek A. Harhala, Karolina Lahutta, Zuzanna Kaźmierczak, Łukasz Łaczmański, Marlena Kłak, Krystyna Dąbrowska

**Affiliations:** ^1^Bacteriophage Laboratory, Institute of Immunology and Experimental Therapy, Polish Academy of Sciences, Wrocław, Poland; ^2^Research and Development Center, Regional Specialist Hospital, Wrocław, Poland

**Keywords:** qPCR, plaque assay, phage, quantitation, antibody, neutralizing, humoral response, immune response

## Abstract

The most common method for phage quantitation is the plaque assay, which relies on phage ability to infect bacteria. However, non-infective phage particles may preserve other biological properties; specifically, they may enter interactions with the immune system of animals and humans. Here, we demonstrate real-time quantitative polymerase chain reaction (qPCR) detection of bacteriophages as an alternative to the plaque assay. The closely related staphylococcal bacteriophages A3R and 676Z and the coliphage T4 were used as model phages. They were tested *in vivo* in mice, *ex vivo* in human sera, and on plastic surfaces designed for ELISAs. T4 phage was injected intravenously into pre-immunized mice. The phage was completely neutralized by specific antibodies within 5 h (0 pfu/ml of serum, as determined by the plaque assay), but it was still detected by qPCR in the amount of approximately 10^7^ pfu/ml of serum. This demonstrates a substantial timelapse between “microbiological disappearance” and true clearance of phage particles from the circulation. In human sera *ex vivo*, qPCR was also able to detect neutralized phage particles that were not detected by the standard plaque assay. The investigated bacteriophages differed considerably in their ability to immobilize on plastic surfaces: this difference was greater than one order of magnitude, as shown by qPCR of phage recovered from plastic plates. The ELISA did not detect differences in phage binding to plates. Major limitations of qPCR are possible inhibitors of the PCR reaction or free phage DNA, which need to be considered in procedures of phage sample preparation for qPCR testing. We propose that phage pharmacokinetic and pharmacodynamic studies should not rely merely on detection of antibacterial activity of a phage. Real-time qPCR can be an alternative for phage detection, especially in immunological studies of bacteriophages. It can also be useful for studies of phage-based drug nanocarriers or biosensors.

## Introduction

Bacteriophages can be used in multiple medical applications (Miȩdzybrodzki et al., 2012; Kutter et al., 2015; Vandenheuvel et al., 2015; Karimi et al., 2016; Saeed et al., 2017), veterinary (Grant et al., 2016), biotechnology (Oślizło et al., 2011; Lee et al., 2012; O’Sullivan et al., 2016), agriculture (Żaczek et al., 2015), or food processing (Endersen et al., 2014). Development of all these phage applications relies on experimental testing with the accurate detection of phage particles in various conditions. Specifically, phage quantitation is the key step in comparisons between different phage strains, different health status of animals or humans, and different experimental design. The most common and prevalent method for phage quantitation is the plaque assay. It simply employs a microbiological culture, where a sensitive bacterial host is cultured with a phage in a double layer plate, eventually allowing for direct counting of plaques as soon as bacteria become visible in the plate (Adams, 1959). There are important limitations of this method. The most important one is the fact that microbiological detection of phage activity does not meet the real count of bacteriophage particles in samples; it in fact allows for testing how many phages were able to infect their host effectively. Phage capability of infection, in turn, may be dependent on a myriad of factors: from simple ion content in the environment and presence of organic compounds or detergents, to the presence of specific antibodies, complement system elements or competitive phages of other types (Ishiguro et al., 1983; Matsushita et al., 2011; Refardt, 2011; Cheng et al., 2013, 2016; Hodyra-Stefaniak et al., 2015; Szermer-Olearnik et al., 2017). Once inactivated, phage cannot be detected by the plaque assay, which makes quantitation of real phage particle content very difficult or even impossible.

Non-infective phage particles may preserve other biological or technological properties that are not related to their ability to infect bacteria. Phage immunoreactivity, i.e., the potency of phage to interact with antibodies and other elements of the immune system, is a phenomenon independent on phage infectivity (Kirsch et al., 2008; Samoylova et al., 2012; Dąbrowska et al., 2014). Most bacteriophages are complex, multi-protein structures where the infection apparatus is only a fraction of the whole particle. Other structural proteins can interact with mammalian system (Dąbrowska et al., 2006, 2007; Barr et al., 2013) regardless phage infectivity. Therefore, phage pharmacokinetics/pharmacodynamics studies should not rely merely on detection of antibacterial activity of a phage.

Techniques that rely on nucleic acid amplification and detection are among the most valuable tools in biological research. Real-time quantitative polymerase chain reaction (qPCR) detection of eukaryotic viruses in environmental and human or animal samples is a standard and commercialized method (Watzinger et al., 2004; Hmaied et al., 2015). Bacteriophages have been quantitatively analyzed and discriminated by real-time qPCR directly in microbiological cultures, and the authors found this method to be a good alternative to the plaque assay (Edelman and Barletta, 2003; Clokie, 2009; Anderson et al., 2011; Refardt, 2012; Dieterle et al., 2016). Real-time PCR has been further demonstrated as applicable for a rapid screening allowing phage detection in food (milk, fruits, vegetables, seafood, meat) (Imamovic and Muniesa, 2011; Flannery et al., 2014; Perrin et al., 2015; Parente et al., 2016; Hartard et al., 2017) and water samples (Farkas et al., 2015; Kunze et al., 2015; Unnithan et al., 2015; Mankiewicz-Boczek et al., 2016) or in feces (Imamovic et al., 2010; Chehoud et al., 2016). However, potential applicability of real-time qPCR for detection of inactivated (non-infective) but still biologically active (e.g., immunoreactive) phage has never been investigated.

Here we propose real-time qPCR as a quantitative method for phage detection in immunological studies of bacteriophages. Specifically, it was tested in comparisons between different phage strains, different immunological statuses of animals or humans, and different experimental designs. Two closely related staphylococcal bacteriophages, A3R and 676Z, together with the coliphage T4, were used as the model phage strains. We assessed and validated a real-time qPCR-based method for immunological studies of samples derived from animals and humans (*in vivo* and *ex vivo* experiments), as well as for optimization of comparative ELISAs of different phages.

## Materials and Methods

### Preparation of Phages T4, A3R, and 676Z and Determination of Phage Titers Using Plaque Assay

T4 phage was purchased from American Type Culture Collection (ATCC, Manassas, VA, United States) and phages A3R and 676Z are part of the IIET Microorganisms Collection (Institute of Immunology and Experimental Therapy, Polish Academy of Sciences, Wroclaw, Poland). Enriched broth cultures of phages were purified by filtration through polysulfone membranes and by fast protein liquid chromatography: gel filtration on Sepharose 4B (Sigma–Aldrich, Poland). The final preparation was dialyzed using 1000 kDa-pore membranes against PBS to remove the bacterial residuals and lipopolysaccharide (LPS), and filtered through 0.22 μm PVDF filters (Millipore, Europe). Prior to dialysis of T4 phage we used LPS-affinity chromatography EndoTrap HD according to the manufacturer’s instructions (Hyglos GmbH, Bernried, Germany) in order to further remove LPS. LPS removal was done by three successive incubations of the preparation with the slurry followed by centrifugations. Each purified phage T4 preparation was tested for phage concentration by determining phage titer after serial dilution with PBS (dilutions from 10^-1^ to 10^-9^), 25 μl of each dilution was spotted on a culture plate pre-covered with susceptible bacteria, two spots for each dilution. The plate was incubated overnight at 37°C to obtain visible plaques. The plaques were counted, mean values of two spots were calculated, and the phage concentration was calculated per milliliter with regard to the dilution and spot volume. For phages A3R and 676Z we used double layer agar plates according to Adams in order to determine phage titer (Adams, 1959).

### Isolation of Genomic DNA from T4, A3R, and 676Z Phages and Preparation of DNA Standards

Phage genomic DNA was isolated using GenElute Mammalian Genomic DNA Miniprep (Sigma–Aldrich, Poznan, Poland). For this procedure we used phage lysates each containing 10^8^ pfu. After incubating samples with DNase and RNase (Sigma–Aldrich, Poland) (50 μg of each, 10 min, 37°C), 40 μg of proteinase K (Sigma–Aldrich, Poland) as well as 100 μl of Resuspension Buffer was added to each sample. Samples were then incubated for 5 min at 70°C. Next, 200 μl of Lysis Solution C was added to the samples and samples were incubated for 10 min at 70°C. Afterward 200 μl of 96% ethanol (Sigma–Aldrich, Poland) was added to each sample. Samples were applied to the columns provided with the kit and prepared in advance using Column Preparation Solution. Following column centrifugation (6500 × *g*, 1 min, room temperature) 500 μl of wash buffer was applied to the columns and the columns were centrifuged (12,000 × *g*, 1 min, room temperature). The washing step was repeated twice. Finally phage genomic DNA was eluted using 60 μl of DNase-free water. DNA was quantified using NanoDrop (Wilmington, DE, United States). Based on this we prepared 10 ng/μl stocks of phage genomic DNA. In order to prepare a standard curve for each phage genomic DNA we used 10 ng/μl stock solutions to prepare the following dilutions of phage genomic DNA: 1, 0.1, and 0.01 ng/μl. For the phages A3R, 676Z, and T4 the highest standard equals approximately 6.5 × 10^7^, 8.6 × 10^7^, and 5 × 10^7^ phage particles per microliter, respectively. Numbers of virus particles were calculated as genomic equivalents. In order to calculate genomic equivalents based on the amount of nanograms of DNA in qPCR samples we used an online calculator (Stothard, 2000) to calculate the molecular weight of a single phage genome from the exact genomic sequences of the bacteriophages (accession numbers for A3R, 676Z, and T4, are JX080301, JX080302, and NC_000866, respectively). The following molecular weights were calculated for single phage DNA molecules: 87108105.24 Da for A3R, 91769200.84 Da for 676Z, and 104340909.87 Da for T4 phage. These molecular weights of phage genomes were used to calculate numbers of single genomic DNA molecules in investigated samples (1 Da weighs approximately 1.67 × 10^-24^ g), resulting in the values called *genomic equivalents*. The standard solutions were used in duplicate to prepare the standard curve for qPCR. For the purpose of absolute quantifications, standard curves were created by plotting quantification cycle (*C*q) values against the number of genomic equivalents.

### Primer Design and qPCR Reaction

The genome sequences of T4, A3R, and 676Z phages were obtained from the GenBank (accession numbers for T4, A3R, and 676Z are NC_000866, JX080301, and JX080302, respectively). Based on the genome sequence, qPCR primers were designed using the Primer-BLAST software at the National Center for Biotechnology Information (National Center for Biotechnology Information, 2017).

To detect T4 phage we used forward primer 5′-ACT GGC CAG GTA TTC GCA-3′ and reverse primer 5′-ATG CTT CTT TAG CAC CGG CA-3′. To detect A3R and 676Z phages the following primers were used: forward primer 5′-TGA AGA AGA CCG TGC AGG ATT-3′ and reverse primer 5′-TCA GAA GGA GCT GAT TGA GCG-3′.

The amount of genomic DNA in each test sample was determined using 5× HOT FIREPolEvaGreen qPCR Mix Plus (Solis BioDyne, Tartu, Estonia) following the manufacturer’s recommendations. Briefly, each PCR reaction contained 1 μl of DNA template and 15 pM of each primer as well as 2 μl of 5× HOT FIREPolEvaGreen qPCR Mix Plus in a final volume of 10 μl. Cycling conditions were as described by 5× HOT FIREPolEvaGreen qPCR Mix Plus’s manufacturer. The amount of phage genomic DNA in test samples was calculated based on the standard curve generated using standard solutions prepared as outlined above. qPCR normalization was performed according to MIQE Guidelines (Bustin et al., 2009).

### ELISA

A MaxiSorp flat-bottom 96-well plate (Nunc, Thermo Scientific, Poznań, Poland) was covered with purified phage preparations obtained by chromatography as described above (100 μl per well, as indicated in the figures) sterilely, at 4°C, overnight. Plates were washed five times with PBS and blocked with five times diluted Superblock (Thermo Fisher Scientific Inc., Rockford, IL, United States) for 1 h (100 μl per well) at room temperature. Blocking solution was removed and the plate was washed five times with PBS with 0.05% Tween 20 (Serva, Heidelberg, Germany). Serum diluted 1:100 was applied to the wells in 100 μl per well. Each sample was processed in duplicate. The plate was incubated at 37°C for 2 h. Plates were washed five times with PBS with 0.05% Tween 20 (Sigma–Aldrich, Poland). Diluted detection antibody was added in the amount of 100 μl per well: peroxidase-conjugated AffiniPure goat anti-mouse IgM (Jackson ImmunoResearch Laboratories, West Grove, PA, United States) or peroxidase-conjugated AffiniPure goat anti-mouse IgG (Jackson ImmunoResearch Laboratories, West Grove, PA, United States) at a final dilution of 200,000. The detection antibody was incubated in the wells for 1 h at room temperature in the dark, removed, and the plate was washed five times with PBS with 0.05% Tween 20. TMB X-Treme (Nordic BioSite AB, Sweden) substrate reagent for peroxidase was used (50 μl) according to the manufacturer’s instructions (ImmunO4, Westminster, MD, United States). Twenty-five microliters of 2 N H_2_SO_4_ (Sigma–Aldrich, Poland) was added to each well without substrate removal, then absorbance was measured at 450 (main reading) and 550 nm (background). The background values were subtracted from the main readings and the average value of each duplicate was calculated. In the case of relative increases of antibody levels OD values are presented.

### Experimental Design

#### Experiment 1: Comparison of qPCR and Plaque Assay Method for T4 Phage Detection in Human Sera *ex Vivo*

In this experiment equal volumes of T4 phage (50 μl, titer 10^7^ pfu/ml) were mixed with equal volumes of human serum samples. We specifically used serum samples from three healthy donors that contained high amounts of antibodies against T4 phage as well as samples from three healthy donors that contained low amounts of such antibodies. The serum samples mixed with T4 phage were incubated for 1 h at 37°C in order to allow for antibody-mediated destruction of T4 phage followed by detection of the amount of live phage in serum samples using the plaque method. In addition we used the qPCR method described above to detect genomic DNA.

#### Experiment 2: Comparison of qPCR and Plaque Assay Method for T4 Phage Detection in Serum of Mice Immunized to T4 Phage

Three BALB/c female mice were injected intraperitoneally on day 0 with 1 × 10^11^ pfu T4 phage/mouse and three BALB/c female mice were injected with an equal volume of sterile PBS. Eleven days after the challenge animals were bled from the tail and serum was separated from the blood by double centrifugation at 2250 × *g*. The serum samples were subjected to the ELISA test in order to determine the amount of antibodies specific for T4 phage belonging to classes IgM and IgG. Thirteen days after the challenge mice were injected i.v. with T4 phage 1 × 10^11^ pfu/mouse determined by the plaque method and bled from the tail 0.5 and 5 h later. Serum was separated as outlined above. A portion of each serum was used to detect the amount of T4 phage via the plaque method, whereas a 50 μl portion of serum was subjected to isolation of viral nucleic acid using a viral RNA and DNA kit (Macherey Nagel, Duren, Germany) according to the protocol outlined by the manufacturer. Obtained DNA was used to detect the amount of T4 phage via the qPCR method.

#### Experiment 3: Quantitation of Phage Particles on Plastic Surfaces

We used dilutions of each phage (T4, A3R, and 676Z) that contained 1 × 10^9^, 5 × 10^9^, and 1 × 10^10^ pfu/ml as determined by the plaque assay, six replicates each. Hundred microliters of each dilution for each phage was used to coat a 96-well Maxisorp plate (Nunc). As a negative control we used PBS solution that was also used as a diluent for phage solutions. The plate was incubated overnight at 4°C. The next day the plate was washed five times with 150 μl of PBS per well and non-specific binding was blocked with fivefold diluted Superblock (Thermo Fisher Scientific Inc., Rockford, IL, United States) for 45 min at room temperature. Then, 10 μg of proteinase K (A&A Biotechnology, Gdynia, Poland) diluted in Tris-EDTA buffer pH 8.0 (final volume 100 μl) was added to each well and the plate was incubated for 4 h at 50°C. After incubation the content of each well was transferred to a single tube. Tubes were incubated for 20 min at 85°C in order to inactivate proteinase K. Following brief centrifugation samples were frozen at -80°C before further use for qPCR reactions as described above.

#### Experiment 4: Comparison of the Amount of Phage Detectable by qPCR and the Relative Signal from the ELISA

In this experiment A3R and 676Z phages at 1 × 10^9^, 5 × 10^9^, and 1 × 10^10^ pfu/ml dilutions as determined by the plaque assay as well as PBS were used in triplicate to coat Maxisorp plates. One plate was processed for qPCR analysis of adhered phage particles as described above (see Experiment 1), and the other identical plate was processed for ELISA. In the ELISA experiment we used mouse serum specific to major capsid protein (AFN38316) as the first antibody source and goat anti-mouse IgG as the detection antibody. The ELISA test was conducted as outlined above.

### Ethics Statement

The female 6- to 8-week-old BALB/c mice were purchased from Mossakowski Medical Research Centre, Polish Academy of Sciences, Warsaw, Poland and kept in specific pathogen-free (SPF) conditions in the Animal Breeding Centre of the IIET. The experiments were approved by the First Local Committee for Experiments with the Use of Laboratory Animals, Wroclaw, Poland (no. 92/2016). The Bioethics Committee of Wrocław Medical University approved obtaining blood samples from healthy donors. All study subjects gave written informed consent for participation in the study.

### Data Analysis and Statistics

Each experiment was repeated at least twice and the results of one experiment are shown. The data are presented as mean ± SE. Statistical analysis was done by one-way ANOVA followed by Tukey’s multiple comparison test with a significance level of *p* = 0.05 or two-tailed unpaired *t*-test with a significance level of *p* = 0.05. The graphs and statistical analysis were performed using GraphPad Prism software.

## Results

### Characterization of qPCR Reaction

**FIGURE 1:**
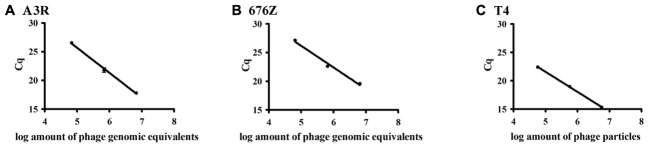
Standard curves representing the correlation between number of phage particles and *C*q values of real-time PCR detection of **(A)** A3R phage, **(B)** 676Z phage, and **(C)** T4 phage (representative examples are presented).

We determined efficiency and specificity of qPCR reactions performed with primers specifically recognizing genomic DNA of staphylococcal phages A3R and 676Z and coliphage T4. In all investigated phages, primers were specific to genes coding for major head proteins. It should be noted that these genes are 100% homologous in A3R and 676Z phages [overall homology of the genomes is 94.6% (Łobocka et al., 2012)]; thus, the same set of primers was used in both phages. Sample standard curves obtained for phages A3R, 676Z, and T4 are presented in **Figure 1**. These curves together with corresponding correlation coefficients (**Table 1**) show a strong correlation between the *C*q value and the genomic equivalents representing numbers of phage particles. The efficiency of amplification reaction for any phage genomic DNA was higher than 75%.

**Table 1:** Correlation coefficients calculated for qPCR reaction (corresponding to **Figure 1**).

PCR reaction specific for	*R*^2^	Slope	Reaction efficiency (%)	*T*_m_
A3R	0.993	-4.083	75.76	77.6
676Z	0.986	-3.77	84.18	78.02
T4	0.999	-3.533	91.89	84.3

**FIGURE 2:**
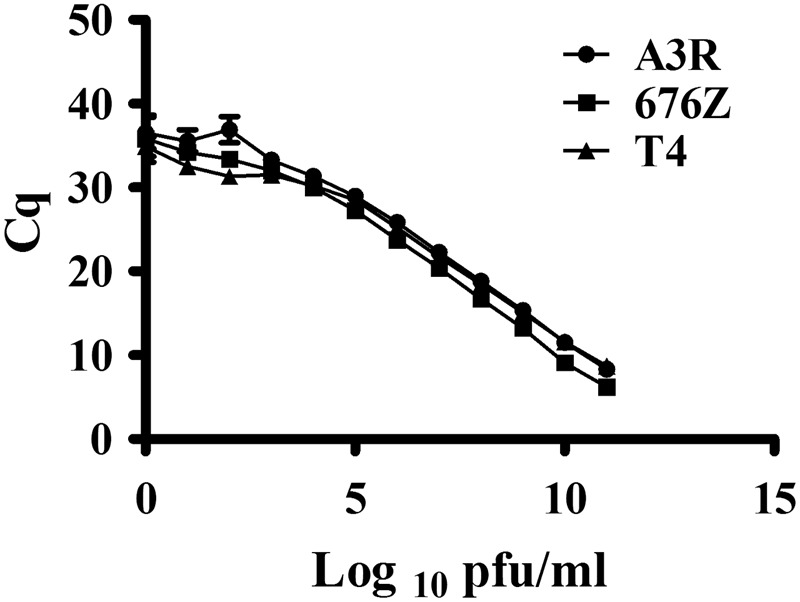
Correlation between *C*q values of qPCR reaction and the amount of phage particles used as a template in qPCR reaction. Three independent serial dilutions (*N* = 3) containing from 0 to 10^11^ pfu/ml of 676Z phage, A3R phage, or T4 phage were used as templates in qPCR reactions. The *X*-axis presents the logarithm of phage titers used as template DNA in the qPCR reaction and the *Y*-axis presents *C*q values of the qPCR reactions.

The sensitivity of phage detection by qPCR reaction was determined by *C*q values of the PCR reaction performed with the phage concentration range from 0 to 10^11^ pfu/ml, for all three investigated phages. We propose the range from 10^3^ to 10^11^ pfu/ml as a reliable correlation between phage concentration and *C*q value; in this range we observed a linear relationship between *C*q values and the number of each phage particles (**Figure 2**). For the lower phage titers *C*q values were characterized by high deviations: in a range between 35.36 ± 0.3 and 35.57 ± 1.32 for A3R phage, between 33.4 ± 0.85 and 34 ± 0.923 for 676Z phage, and 31.32 ± 0.69 and 32.57 ± 0.064 for T4 phage.

### Comparison of qPCR and Plaque Assay Method for T4 Phage Detection in Serum of Mice Immunized to T4 Phage

**FIGURE 3:**
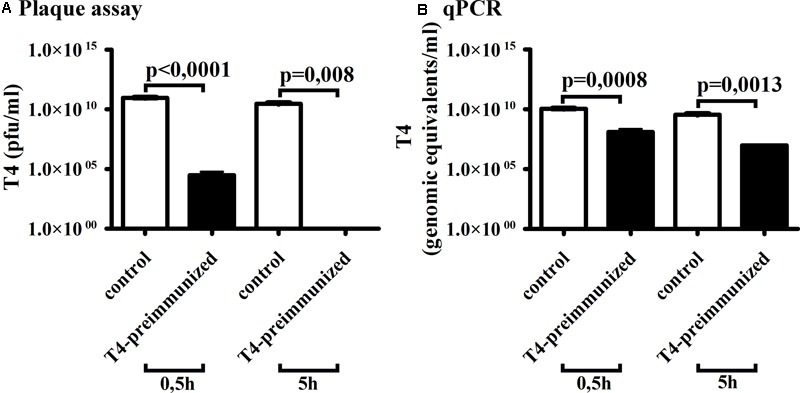
Comparison of qPCR and plaque assay method for T4 phage detection in serum of mice immunized to T4 phage. Control and pre-immunized BALB/c mice were injected i.v. with T4 phage 10^11^ pfu/mouse, murine sera were collected 0.5 and 5 h after and phage concentration in the sera was tested by **(A)** plaque assay method or **(B)** qPCR method.

Optimized qPCR detection of phage (see above) was used to detect the phage in *in vivo* model with animals eliciting phage-neutralizing antibodies. Mice were challenged with T4 to develop a specific antibody response as previously described (Dąbrowska et al., 2014); a high antibody level was confirmed by ELISA (**Supplementary Figure S1**). The mice specifically immunized to T4 phage and control mice were injected intravenously with the phage; phage concentration in blood serum was assessed 0.5 and 5 h after injection by the plaque assay or by qPCR. The amounts of phage detected in murine sera are compared in **Figure 3**. Both methods showed that the presence of specific antibodies results in a decrease of phage concentration in animals’ sera, but there was a striking difference in the demonstrated levels of the decrease. Half an hour after phage administration to mice, the difference between phage concentration in phage-immunized mice and control mice as detected by the plaque assay was more than five orders of magnitude, but only two orders of magnitude by qPCR. Five hours after phage administration to mice, this difference as detected by the plaque assay was more than 10 orders of magnitude (no active phage was detected in immunized mice) but less than three orders of magnitude by qPCR (still approximately 10^7^ genomic equivalents/ml of phage particles detected). This demonstrates that the phage, even when neutralized in terms of antibacterial activity, can still be present in the circulation in high amounts and it can be detected and quantitatively assessed by qPCR.

### Comparison of qPCR and Plaque Assay Method for T4 Phage Detection in Human Sera *ex Vivo*

**FIGURE 4:**
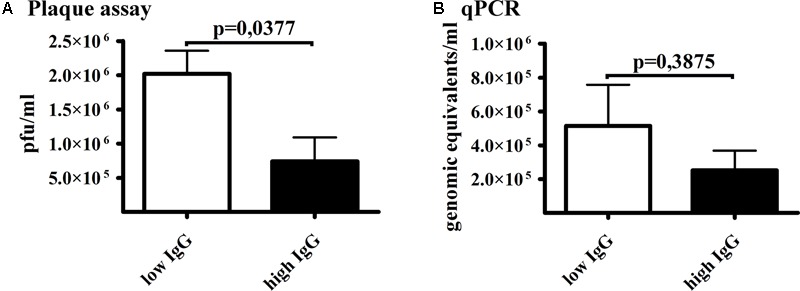
Comparison of qPCR and plaque assay method for T4 phage detection in human sera *ex vivo*. T4 phage 10^7^ pfu/ml was incubated for 1 h with human sera (1:1); the sera had either a high or low concentration of T4 phage-neutralizing antibodies. After the incubation samples were directly used for testing phage concentration by **(A)** plaque assay method or **(B)** qPCR method.

The potency of qPCR to detect immunologically inactivated phage was further verified in human sera *ex vivo*. T4 bacteriophage was incubated with human sera, both with those containing a high concentration of phage-specific antibodies (positive sera) and with those with a low content of phage-specific IgG (negative sera); a previously described collection of positive and negative sera was used (Dąbrowska et al., 2014). After incubation, the phage was detected either by plaque assay or by qPCR and the results were compared. The plaque assay revealed statistically significantly lower (70% decrease) activity of phage after incubation with positive sera than that after incubation with negative sera (*p* = 0.037). In the qPCR method the amount of phage detected in both groups was not statistically different (*p* = 0.39) (**Figure 4**). This is in line with the proposition of qPCR as a method for detection of phage whose antibacterial activity has been neutralized.

### Quantitation of Phage Particles on Plastic Surfaces

Due to the devastating effect that antibodies may exert on phage activity, probably the major area of immunological studies of bacteriophages is identification of phage-specific antibodies. Comparative studies of bacteriophages as different antigens by ELISA need to include quantitation of phage particles immobilized on ELISA plates. Here, quantitation of T4, A3R, and 676Z phage particles adhering to 96-well plates was conducted by qPCR, using the developed standard curves (see section Characterization of qPCR Reaction).

**FIGURE 5:**
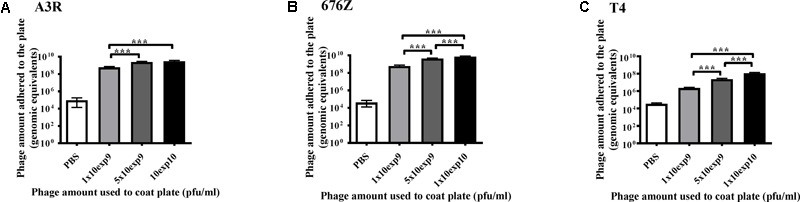
Quantitation of phage particles on plastic surfaces by RT-qPCR. A standard ELISA plate was covered with three dilutions of A3R **(A)**, 676Z **(B)**, or T4 **(C)** phage. The amount of phage used to coat the plate was determined by plaque assay and it is indicated on the *X*-axis of each graph. The amount of genomic equivalents recovered from the plate was determined by qPCR reaction and it is presented on the *Y*-axis. ^∗∗∗^*p* < 0.001.

In general, all investigated bacteriophages were detectable by qPCR when immobilized on ELISA plates. We further observed that the amount of immobilized phage correlated positively with concentration of the phage that was used for incubation with the plate (**Figure 5**). Comparison of the three investigated phages revealed that their individual affinity to plastic surfaces was markedly different. Incubation with the highest phage concentration (1 × 10^10^ pfu/ml) allowed for the following approximated amounts of recovered bacteriophages (as genome equivalents): 1 × 10^8^ for T4, 3 × 10^9^ for A3R, and 6 × 10^9^ for 676Z (**Figure 5**).

**FIGURE 6:**
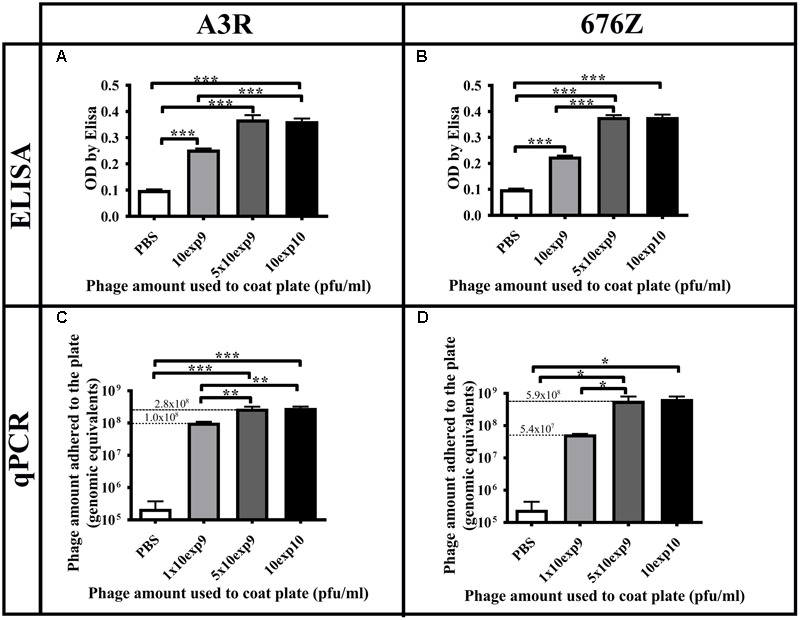
Comparison of the amount of phage detectable by qPCR and the relative signal from ELISA. ELISA plates were coated with three dilutions of A3R or 676Z phage. The plates were processed either for **(A,B)** immunodetection of phage by ELISA with major capsid protein-specific antibodies, or **(C,D)** for qPCR detection of phage immobilized on the plate, with PCR primers specific to the genes coding for major capsid proteins. ^∗^*p* < 0.05, ^∗∗^*p* < 0.01, ^∗∗∗^*p* < 0.001.

We further compared the amount of phage detectable by qPCR and the relative signal from the ELISA, to assess the reliability of ELISA for comparisons between phages. ELISA plates covered with two very similar bacteriophages, A3R and 676Z, were processed either for adhering phage quantitation by qPCR or for immunodetection of phage by major capsid protein-specific antibody (ELISA). Although the qPCR method revealed approximately two times more 676Z phage adhering to the ELISA plate (in comparison to A3R phage), no differences were detected by ELISA (**Figure 6**).

Estimation of the efficiency of phage immobilization in the well was calculated from the total amount of phage DNA in the solution that was applied to the wells and from the amount of phage DNA recovered from the wells. It revealed that when 1 × 10^10^ pfu/ml solutions of phages A3R and 676Z were applied to the wells we recovered 4.5 and 9.67%, respectively.

## Discussion

In this work we investigated qPCR as a useful alternative for phage detection, in comparison to standard methods based on phage cultures with bacteria. We focused on immunological studies of bacteriophages, since antibodies are probably the most efficient biological factors able to neutralize phages. We demonstrated that the phage, even when neutralized in terms of antibacterial activity, can still be present in the circulation in high amounts and it can be detected and quantitatively assessed by qPCR (**Figure 3**). This implies that the phage may exert biological effects much longer than it can be detected by standard microbiological methods. Phage effects that are independent of its antibacterial activity may apply to phage use as drug nanocarriers (Karimi et al., 2016; Bashari et al., 2017), vaccine platforms (Aghebati-Maleki et al., 2016; Pires et al., 2016; Tao et al., 2016), as well as to possible immunomodulation exerted by the phage (Barr, 2017; Górski et al., 2017). Our observations demonstrate that the time lapse between “microbiological disappearance” and true clearance of phage particles from the circulation can be substantial. This particularly applies to common phages that may run up against a frequent and strong anti-phage immune response in the population, such as T4 (Dąbrowska et al., 2014).

A further consequence of the strong influence of antibodies on the results of microbiological detection of phages is the fact that standard attempts to detect phages in human and animal sera can be unsuccessful even when the phages are highly represented there. Phage translocation and “phagoviremia” has been postulated as an important physiological phenomenon (Górski et al., 2006), although not demonstrated yet. There are still not enough data to assess levels of immunization of humans and animals to phages naturally belonging to their gut- (or other) microbiota. However, a model phage was demonstrated as able to induce a specific systemic response and a high serum level of specific IgG (Majewska et al., 2015). Assuming that phages of the microbiome eventually induce antibody production, it may be impossible to detect true phage translocation to the circulation only by microbiological methods. We propose the qPCR method as an appropriate and reliable method for studies of phage translocation to blood. This method can also be applied in other experiments that require detection of a “neutralized” phage, which is merely a phage that is not active against bacteria. Notably, qPCR has already been applied for phage detection in other (than phage neutralizing) conditions: in phage cultures (Edelman and Barletta, 2003; Clokie, 2009; Anderson et al., 2011; Refardt, 2012; Dieterle et al., 2016), food (Imamovic and Muniesa, 2011; Flannery et al., 2014; Perrin et al., 2015; Parente et al., 2016; Hartard et al., 2017; Muhammed et al., 2017), environmental samples (Farkas et al., 2015; Kunze et al., 2015; Unnithan et al., 2015; Mankiewicz-Boczek et al., 2016), and in feces (Imamovic et al., 2010; Chehoud et al., 2016), where some interference from antibodies cannot be excluded (Majewska et al., 2015).

We have also shown that different bacteriophages may differ strongly in their ability to immobilize on plastic surfaces. Here, we report more than one order of magnitude difference between phage recovery from plastic that was covered by different phages in the same conditions (**Figure 5**). This should be considered in any comparative studies of bacteriophages that include phage immobilization on various surfaces, e.g., in biosensor constructing or in the ELISA assay for phage immune-reactivity. As demonstrated by comparison of the ELISA signal to qPCR phage detection, differences in phage ability to adhere to a plastic surface can be missed by immunodetection (**Figure 5**). Notably, the two staphylococcal bacteriophages investigated in this work are highly similar. They have identical genes coding for major capsid proteins (the ones detected by qPCR here) (Łobocka et al., 2012), but minor structural elements of their virions differ. Eventually, their adherence to plastic also differs as much as twofold (**Figures 5, 6**).

Quantitative analysis of phage by real-time PCR as a method has a few limitations that should be considered when planning its use. First, although qPCR can detect even those phage particles that are not able to infect bacteria, it is sensitive to inhibitors of the PCR reaction. Thus, in conditions where these inhibitors cannot be removed, phage detection may be more efficient in the plaque assay that in qPCR. For example, our *post hoc* analysis indicated that the animals in this study were challenged with 5.8 × 10^11^ pfu/ml of T4 as measured by plaque assay and 1.56 × 10^11^ of T4 as measured by qPCR. We also observed an inhibitory effect of raw human sera when applied into the composition of the qPCR reaction (data not shown). Thus, we isolated phage DNA from murine sera by a standard isolation kit to compare phage circulation between immunized and non-immunized mice. This approach turned out to be appropriate to overcome the problem of PCR reaction inhibitors in serum samples. The second considerable requirement for experiments employing qPCR is to use phage preparations without free phage DNA. In the qPCR method, free phage DNA present in phage solution can easily produce a false positive signal mimicking phage particles. It means that production of phage preparations, including often complex procedures of phage purification, need to be gentle enough not to destroy phage particles releasing phage nucleic acids.

### Concluding Remarks

This study demonstrates a substantial time lapse between inactivation of antibacterial activity and true clearance of phage particles from the circulation of pre-immunized individuals. qPCR allows for detection of inactivated bacteriophages that cannot be detected by plaque assay. Further, qPCR demonstrated marked differences in the ability of investigated bacteriophages to immobilize on plastic surfaces, while the ELISA did not detect differences in phage binding to plates. We propose that phage pharmacokinetic and pharmacodynamic studies should not rely merely on detection of antibacterial activity of a phage; real-time qPCR can be an extension for phage detection methods.

## Author Contributions

AK performed most of the experiments, analyzed the results, and participated in writing the manuscript. AZ, DL, JM, MH, KL, MK, and ZK participated in experimental work. ŁŁ participated in experimental design, data analysis, and reviewed the manuscript. KD conceived and designed the experiments, participated in experimental work and in data analysis, and wrote the paper.

## Conflict of Interest Statement

The authors declare that the research was conducted in the absence of any commercial or financial relationships that could be construed as a potential conflict of interest.

## Supplementary Material

FIGURE S1 T4-specific antibody detection in mice pre-immunized with T4. Significant induction of serum IgM and IgG specific for T4 phage was detected by ELISA (IgM level: T4-challenged mice vs. PBS control, *p* = 0.005; IgG level: T4-challenged mice vs. PBS control, *p* < 0.0001).
