# Supplementary figures and images for: Real-Time qPCR as a Method for Detection of Antibody-Neutralized Phage Particles
Source: Front Microbiol. 2017 Nov 6;8:2170. doi: 10.3389/fmicb.2017.02170 (PMC5672142; doi:10.3389/fmicb.2017.02170)

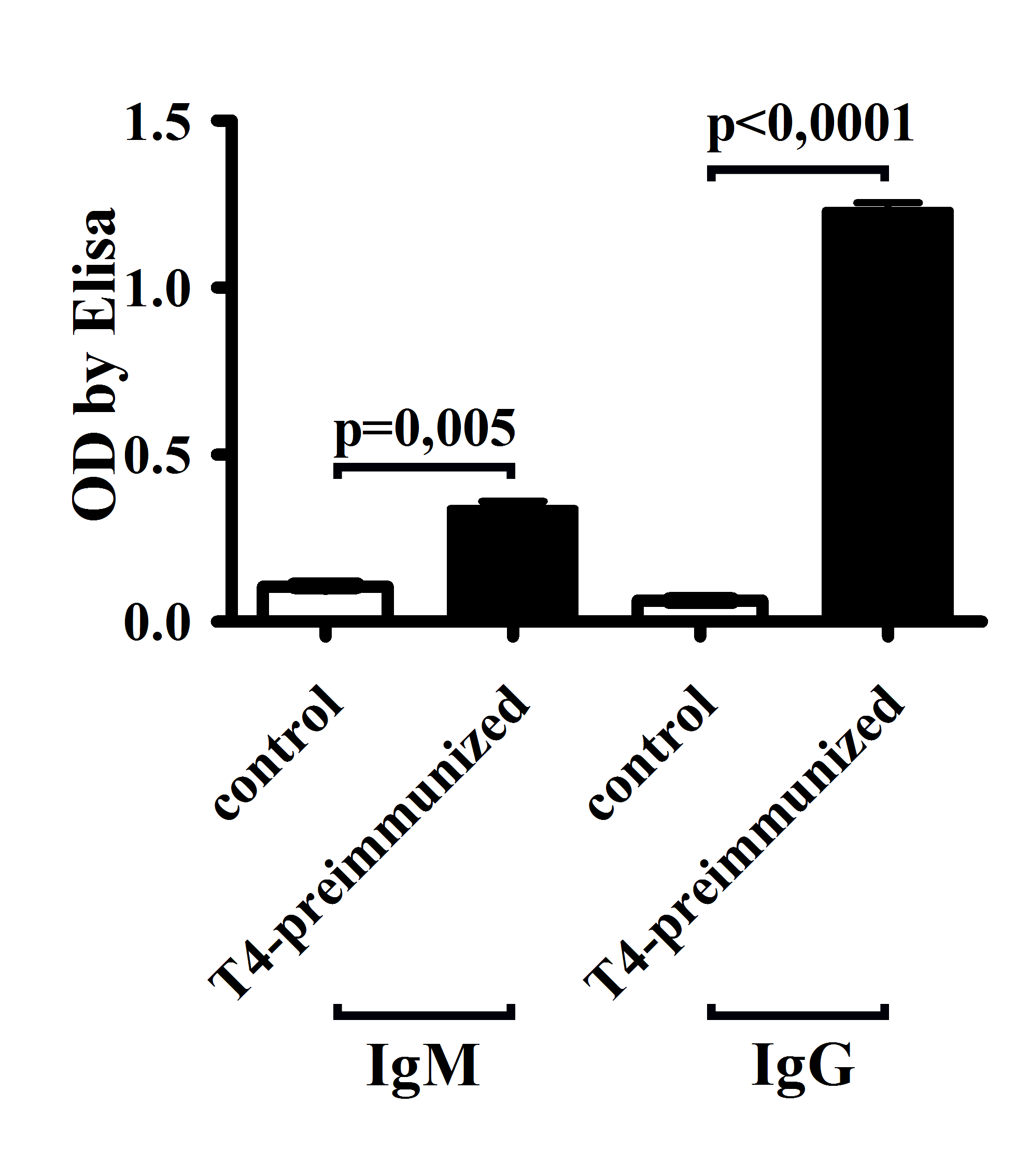

Supplement: FIGURE S1 — T4-specific antibody detection in mice pre-immunized with T4. Significant induction of serum IgM and IgG specific for T4 phage was detected by ELISA (IgM level: T4-challenged mice vs. PBS control, p = 0.005; IgG level: T4-challenged mice vs. PBS control, p < 0.0001). [file Image_1.TIF]
